# Measuring and understanding motivation among community health workers in rural health facilities in India-a mixed method study

**DOI:** 10.1186/s12913-016-1614-0

**Published:** 2016-08-09

**Authors:** Jaya Prasad Tripathy, Sonu Goel, Ajay M. V. Kumar

**Affiliations:** 1 International Union Against Tuberculosis and Lung Disease, The Union South East Asia Office, New Delhi, 110016 India; 2 Department of Community Medicine, School of Public Health, Post Graduate Institute of Medical Education and Research, Chandigarh, 160012 India

**Keywords:** Motivation, Health workers, Rural, India, Job satisfaction

## Abstract

**Background:**

Motivated human resource is the key to improve health system performance and retention of health workers. There is scanty literature on measuring motivation of health workers in India. Thus, the objective of this study was to measure and identify important aspects of health workers’ motivation in North India.

**Methods:**

A mixed method study design was adopted. Under the quantitative component, we interviewed randomly selected 62 community health workers (CHWs) in 18 sub-centres in two blocks of District Ambala, Haryana, India using a structured motivation scale. In-depth interviews were also carried out with 18 CHWs to explore the sources of motivation.

**Results:**

The age of respondents and training in the past 12 months were found to be significantly associated with motivation. Job burnout, poor personal health, job insecurity and less career development opportunities were the individual level de-motivators, whereas not being able to fulfil family roles and poor supportive supervision were identified as environmental factors for poor motivation. Love for work, and financial incentives were individual level motivators, while community support and recognition, organizational commitment and pride, regular training were identified as environmental level motivators.

**Conclusion:**

Non-financial motivators such as interpersonal relations, family support, skill and career development opportunities require more attention. Regular need-based training is essential to maintain high levels of motivation.

**Electronic supplementary material:**

The online version of this article (doi:10.1186/s12913-016-1614-0) contains supplementary material, which is available to authorized users.

## Background

Human resource is the backbone of the health system and accounts for the largest share in health expenditures. Human resource in the health sector has been grappling with perennial problems such as staffing shortages, poor job conditions, low remuneration and high turnover [1, 2].

Governments have been recruiting and training community health workers (CHWs are defined by World Health Organization (WHO) as members of, selected by and answerable to the communities where they work, supported by the health system and receiving less training than formally trained health workers) for decades, following the 1978 Declaration of Alma-Ata [3], to provide primary healthcare to all. There is conclusive evidence about the success of CHWs in addressing community health issues. CHWs have been shown to contribute to reductions in child morbidity and mortality, encourage immunization uptake, promote breastfeeding, and improve outcomes among tuberculosis patients and children suffering from acute respiratory infection or malaria [4, 5].

Motivation is defined as an individual's degree of willingness to exert and maintain an effort towards attaining organizational goal [6]. A key constraint in achieving the Millennium Development Goals (MDGs) was the absence of a properly trained and motivated workforce [7]. Now, with the Sustainable Development Goals (SDGs) in place, the presence of skilled, motivated staff is critical towards improving health system performance and achieving the targets [8]. Poor motivation, on the other hand, leads to absenteeism, high turnover, poor work performance and shirking of responsibilities [9].

In the twenty-first century, it is the human asset, not the fixed asset that will make the difference for successful organizations. It is therefore important to assess the levels of motivation of manpower and understand the factors associated with poor motivation. Efforts to improve health worker motivation have traditionally focused on financial incentives, including pay-for-performance and incentives to work in underserved and hazardous areas [9–12]. However, according to The Joint Learning Initiative Report, motivating factors differ widely with factors other than remuneration having higher importance [13]. To maximize performance, health workers must work in environments with incentives in place that reward high quality performance. The present study was planned to help us understand the domains of motivation in order to design the health system with appropriate package of incentives, both financial and non-financial.

India, like the world over faces various human resource issues such as acute shortage of skilled manpower, low motivation, poor retention, inequitable distribution etc [14, 15]. Despite efforts to address these issues, the health system has failed to attract and retain health-care providers in rural areas. Attention to motivation is important because factors that influence the performance of health-care workers and the health sector are a function of motivation [16]. Although there are some studies in India exploring levels of motivation and factors associated with it, studies on this subject matter remain limited. Published literature in India have focused on specific cadre of health workers such as ASHAs [Accredited Social Health Activists], doctors or private sector health personnel or have used either qualitative or quantitative method [17–20]. Therefore, we sought to measure and identify sources of motivation and demotivation among rural community health workers (CHWs) working at various levels of the health system in the state of Haryana in North India using a mixed method approach.

## Methods

### Study design

A mixed method study design was used. The quantitative component was followed by a qualitative component for in-depth and comprehensive understanding of the factors under interest. Specific conceptual frameworks were used to analyse the data collected and generate themes and sub-themes to understand the domains of motivation.

### General setting

Haryana is one of the wealthier states of India with the second highest per capita income in the country [21]. With a population of more than 25 million [22], the state is divided into 21 districts. District Ambala has a population of around 1.1 million sprawling across 1574 km [2] and population density of 720/sq.km. With a high literacy rate of 82 % and nearly 50 % of population in urban areas, Ambala is one of the progressive districts in the state [23].

### Quantitative part

The study was carried out in two administrative blocks of Ambala district, namely Shahzadpur and Ambala in year 2013 which have a total of two Community Health Centres (CHCs) and nine Primary Health Centres (PHCs). Two sub-centres (*n* = 18) were selected randomly from each PHC. From each sub centre, we randomly selected and interviewed at least three health workers [one Auxiliary Nurse Midwife (ANM), one Accredited Social Health Activist (ASHA) and one Anganwadi Worker (AWW)]. In some sub-centres (*n* = 8) with a large population, two ASHAs were selected. Every village in India has a trained female community health activist called ASHA. She works as an interface between the community and the public health system. She creates awareness on health issues, promotes good health practices, mobilizes and facilitates community in accessing health services. ANM is posted at a sub-centre level which is a health facility for every 5000 population and provides basic promotive, preventive and curative services to the community. Each ANM supervises 4–5 ASHA workers. Both receive performance based incentives for the services rendered. Every village has an Anganwadi Centre (AWC) which provides services such as supplementary nutrition to children and pregnant women, non-formal pre-school education, growth monitoring and health education. These services at an AWC are delivered by an AWW who is an honorary worker belonging to the same locality.

To measure motivation, we used a tool adapted from a motivation construct developed by Mbindyo et al. which was adapted from Bennet et al [16, 24]. The tool was revised based on a comprehensive review of literature related to the constructs of motivation among health care workers and expert opinion. The revised tool was pretested among six health care workers and two health supervisors. Based on their responses the tool was again modified. The final pretested tool had 23 items, with answers given on an agreement scale of 1 to 4 (1 = strong disagreement, 4 = strong agreement). Reverse coding was done for negative questions before analysis. The scale for negatively worded question was 1 (strong agreement) to 4 (strong disagreement). The tool had eight major constructs: general motivation, burnout, job satisfaction, intrinsic job satisfaction, organizational commitment, conscientiousness, timeliness and personal issues. The items with a median score of more than 2 were considered as important sources of motivation, while those with score ≤2 were considered sources of demotivation Additional file 1.

All respondents were informed about the purpose of the survey and included in the study after obtaining written consent. The tool was administered by a trained interviewer. Data were entered into Microsoft Excel and exported to EpiData (version 2.2.2.183) for analysis. Motivation scores were summarized by median score. Mean motivation scores were compared across various sub groups using ANOVA considering the normality of the distribution of the data. The total motivation score for each respondent was computed by adding the scores against all 23 items in the questionnaire. The minimum and maximum possible score of the tool was 23 and 92 respectively.

### Qualitative part

In-depth interviews (IDIs) were carried out with 18 CHWs (no refusals) purposively chosen among those who participated in the quantitative part of the study and were found to be expressive. The interviews were conducted by the principal author (JPT), a male researcher who is well trained in qualitative research (MD in Community Medicine) and having more than 4 years of work experience in the study area. During data collection, he was working as a Senior Resident at the Department of Community in a premier tertiary medical college in North India. The interviewer (JPT) was temporarily posted (for 6 months) in the field practice area of the department where the study was conducted. He is well versed in local language and the interviews were conducted in local language. The IDIs were conducted face-to-face at a time and place convenient to the participants using an interview guide. The interviews were conducted at their workplace i.e. health centres where they are posted. The pilot-tested interview guide included questions exploring motivators and de-motivators at the individual, community and health system level. Each interview took around 20 min. Participants were informed of the purpose of the study. Only the participant and the researcher were present during the interview. After the interview, the summary of the interviews was read back to the participants to ensure participant validation.

The principal author noted down the proceedings during the interviews and transcribed on the day of the interview. No audio and video recording was done. Content analysis of the transcripts was performed manually [25]. This was then reviewed by the second author, who was also trained in qualitative research in order to reduce bias and interpretive credibility. A descriptive thematic analysis was done using a framework approach. Coding and theme generation were done by standard procedures and in consensus [26]. Any difference between the two authors was resolved by discussion. Codes generated from the interview data were systematically applied to identify themes [21]. The findings have been reported in accordance with the ‘Consolidated Criteria for Reporting Qualitative Research’ [27]. Ethics approval was obtained from the Institute Ethics Committee, Post Graduate Institute of Medical Education and Research, Chandigarh, India. Written informed consent was obtained from the participants before any interview.

### Conceptual framework of motivation

We presented the results of our study on the basis of two theoretical models of motivation. The two models: Community Health Workers Motivation Framework & Existence Relatedness Growth Model of motivation tried to relate the factors underpinning motivation of health workers.

### Community health workers motivation framework

**Fig. 1:**
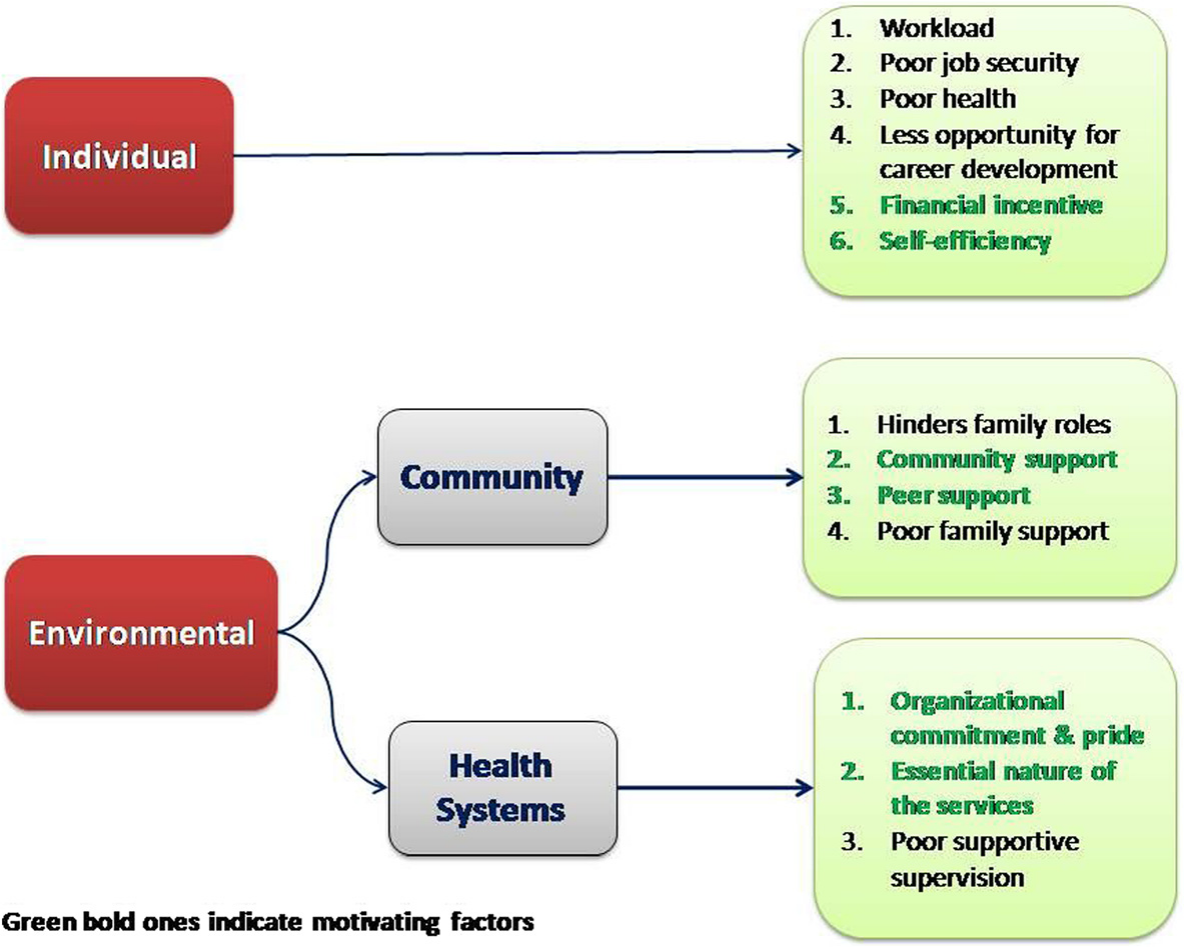
Understanding motivation of community health workers in India - a model based approach

We used a Community Health Workers Motivation Framework by Gopalan et al [19] to understand the aspects of motivation among health workers. The sources of motivation were broadly classified into individual and environmental. The latter was further divided into health system and community level factors. Minor themes (motivators and de-motivators) were identified under the above broad classifications by content analysis of the qualitative data (Fig. 1).

### ERG (Existence Relatedness Growth) model of motivation [28]

**Fig. 2:**
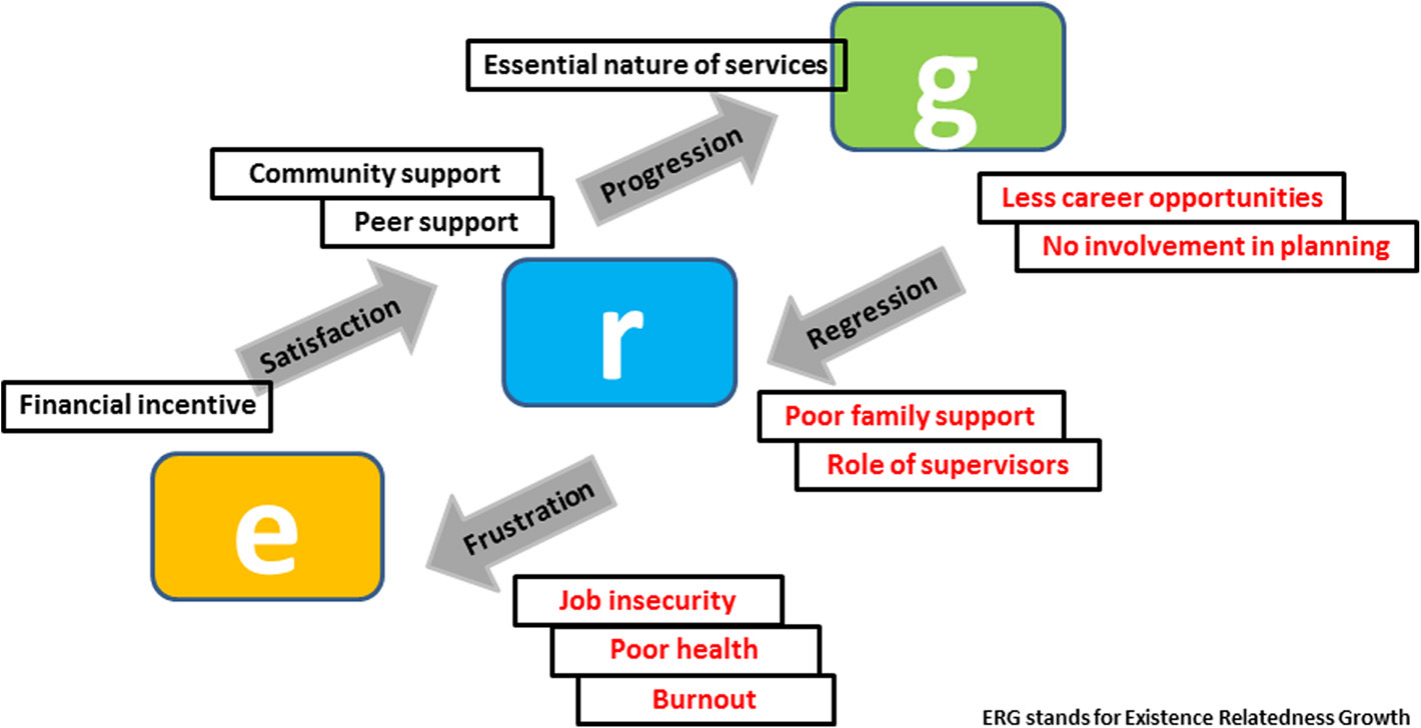
ERG Theory of motivation - looking for a solution

ERG theory holds that an individual has three sets of basic needs: existence, relatedness, and growth. The individual after failing in his attempts to satisfy his higher level needs will return to lower-level needs, thereby breeding frustration which will lead to regression. The theory states that if growth needs are not satisfied, relatedness needs emerge as a major motivating force. In the present context, poor opportunities for development of career and skills and minimal involvement in planning hamper their growth needs. Although support from the community and peers somewhat satisfy their relatedness needs, but support from family and supervisors is what they still look for. Benefits to the family in terms of health benefits and improving the quality of supervision with more emphasis on supportive supervision can to some extent fulfill their relatedness needs. We also have to redirect our attention towards their basic existence needs like health, working conditions and work overload (Fig. 2).

## Results

### Quantitative results

Table 1 shows the basic demographic characteristics of study participants. Most of the health workers were females (94 %) belonging to the age group 40–49 (39 %). There were equal number of ASHAs and AWWs (22, 35 %) and 18 (30 %) ANMs. About half of the respondents had more than 5 years of experience. Two-third of the respondents reported having not attended any training in the preceding 12 months.

**Table 1:** Demographic characteristics of community health workers in District Ambala, Haryana, India, 2013

Variables	Number	Percent
Sex		
Male	4	6.5
Female	58	93.5
Age group in years		
20 to 29	13	21.0
30 to 39	18	29.0
40 to 49	24	38.7
> 49	7	11.3
Type of health worker		
ANM	18	29.0
ASHA	22	35.5
AWW	22	35.5
Time in post		
< 1 year	12	19.4
1–5 years	21	33.9
> 5 years	29	46.7
Received training past 12 months		
Yes	22	35.5
No	40	64.5

A total of 62 health workers completed the motivation assessment scale and none of the health workers refused to participate, giving a response rate of 100 %. The median response score for each item is given in Table 2. Overall, the median (IQR) motivation score of health workers was 61 (58–64) out of a maximum score of 92. The score was highest on the general motivation, intrinsic job satisfaction, organizational commitment and self-efficacy domains with a median score of 3.0 out of total score of 4.0. Job burnout, job insecurity, less opportunities for career development, not being able to fulfil family roles, poor personal health and poor supervision mechanism were some of the de-motivators whereas organizational commitment and pride, self-efficiency, essential nature of services and financial incentive were the key drivers (Table 2).

**Table 2:** Median item-wise motivation scores of community health workers in District Ambala, Haryana, India, 2013

Category	Description of item	Score (1–4)
General Motivation	I feel motivated to work hard	3.0
	Only do this job to get paid	3.0
	I do this job as it provides long-term security for me	1.0
	Overall median domain score	3.0/4.0
Burnout	^a^I feel emotionally drained at the end of the day	2.0
	^a^Sometimes when I get up in the morning, I dread having to face another day at work	2.0
	Overall median domain score	2.0/4.0
Job satisfaction	Overall, I am very satisfied with my job	3.0
	I am satisfied with my colleagues in my work	3.0
	I am satisfied with my supervisor	2.0
	Overall median domain score	2.0/4.0
Intrinsic job satisfaction	I am satisfied with the health services being provided by me	4.0
	I feel that the services being provided by me are essential	4.0
	I get ample opportunities for career and skill development	2.0
	Overall median domain score	3.0/4.0
Organization commitment	I am proud to be working for this health facility	3.0
	I feel very committed to this health facility	4.0
	This health facility really inspires me to do my very best on the job	3.0
	Overall median domain score	3.0/4.0
Conscientiousness and self-efficacy	I can rely on my colleagues at work	3.0
	I always complete my tasks efficiently and correctly	3.0
	Do things that need doing without being asked or told	3.0
	Overall median domain score	3.0/4.0
Timeliness	I am punctual about coming to work	3.0
	^a^I am often absent from work	3.0
	It is not a problem if I sometimes come late for work/on leave	3.0
	Overall median domain score	3.0/4.0
Personal issues	^a^I suffer from health related problems due to the work profile	2.0
	^a^I feel difficulty in doing field activities	3.0
	^a^My work affects my duties towards my family	2.0
	Overall median domain score	2.0/4.0

^a^The scale for these negatively worded questions was reverse coded so that 1 was ‘strong agreement’ and 4 ‘strong disagreement’. Thus, a high score shows disagreement with a negative statement and is therefore suggestive of higher motivation

**Fig. 3:**
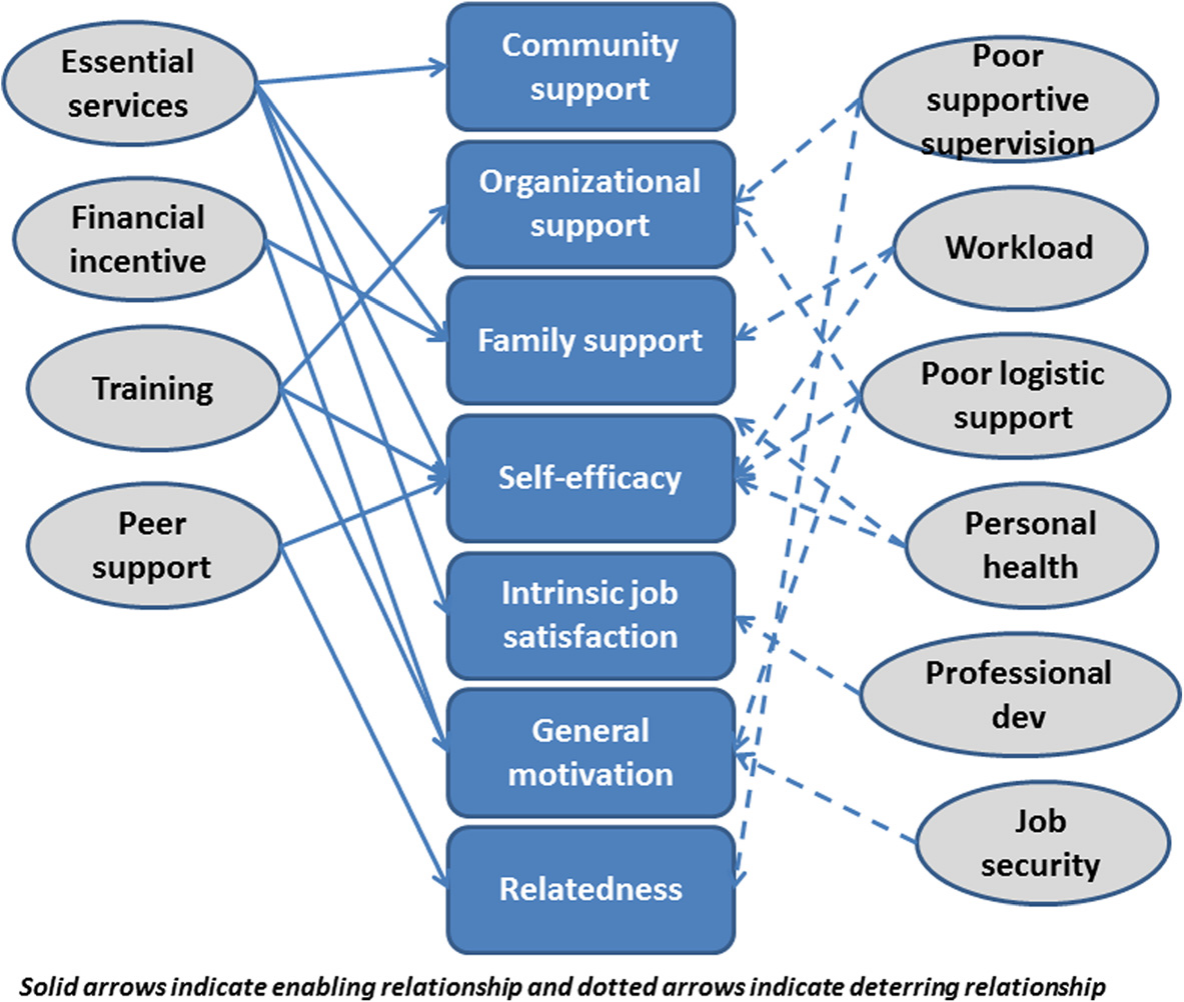
Interlinking relationships between factors affecting community health workers’ motivation

Age and training in the past 12 months were found to be significantly associated with motivation scores (Table 3). Figure 3 shows various factors affecting community health workers motivation and their interlinking relationships.

**Table 3:** Determinants of motivation among community health workers in District Ambala, Haryana, India, 2013

Variables	Number	Mean motivation score	*p*-value
Sex			
Male	4	58.2	0.25
Female	58	61.4	
Age group in years			
20 to 29	13	63.2	0.01
30 to 39	18	63.2	
40 to 49	24	59.6	
>49	7	57.7	
Type of health worker			
ANM	18	62.1	0.53
ASHA	22	61.4	
AWW	22	60.2	
Received training past 12 months			
Yes	22	63.0	0.03
No	40	60.1

### Qualitative results

A total of 18 CHWs were interviewed. All of them were females in the age range 24–50 years. A mix of ANMs (09), ASHAs (05) and AWWs (04) were interviewed.

### Motivating factors

#### Individual level

Individual level factor which motivates CHWs is love for the nature of work and the appreciation received for the service rendered to the community.

*“I love my work because I serve my community and people like me for that. The community looks up to me during illnesses or getting health related information.” –ANM (12)*

The CHWs loved the nature of the job because they are of some help to the community which is appreciated by the family and the community. Essential nature of the services being provided keeps them inspired. People in the community respect them for the service they render to the community. Community service is considered a source of personal pride. The CHWs also feel inspired by the changes they see in the community as a result of their work. One of the CHWs also gave the example of more women going for an institutional delivery than before in their field area which is quite encouraging.

#### Community level

The support and recognition they get from the community where they work is a community level motivator. This support provides an enabling environment for work.

*“We get a lot of support from the villagers during our field visit. People greet us warmly, offer us drinks, food and respond to us very nicely. They accept me because they know me and they appreciate my work”- ASHA (06)*

Good support from the peers and community is what gets them going. Most CHWs reported a positive reception by community members. They gain goodwill and recognition from the community where they work. The community acknowledges their contribution in improving the general health awareness and practices. However, few CHWs reported negative reactions from the community while disseminating messages pertaining to sensitive issues like contraception, HIV etc.

#### Health system level

A healthy organizational environment in terms of supportive colleagues and regular training are the health system motivators.

*“We have a good working and personal relations with our colleagues at the facility, we work together and help each other out.”- ANM (31)*

The health workers reported favorable organizational climate to enhance performance motivation. There is good peer support at the workplace.

*“Training is necessary to keep us updated about new diseases and respond to the queries of the community” – ASHA (44)*

CHWs reported that regular training is necessary to gain new knowledge in view of emerging health issues. The CHWs felt empowered through the acquisition of knowledge and skills on newer issues in community health through training. They were interested in learning and gaining new health related information so that they remain well informed to help their families and the community. Another CHW noted that training keeps alive the excitement to work.

### De-motivating factors

#### Individual level

At the individual level, de-motivators were job related and personal factors. Job related de-motivators included job burnout, inadequate remuneration, poor career development opportunities and security concerns whereas poor personal factor was among the personal factors.

*“Apart from the routine activities, we are asked to attend to any team that visits, rallies, camps, special activities, surveys by various agencies etc. We get very less time for our routine work.”- AWW (17)*

The CHWs were very vocal in saying that they do not have much time for routine activities. They are involved in multiple activities other than their routine stuff like accompanying external agencies during their field visit, organizing rallies, camps, surveys and meetings, assisting in research work for other agencies. Travel takes a lot of their time. There is already a lot of workload. To add to it, whenever new a program/guideline is launched there is more work which hampers their existing routine work. Thus, job burnout and fewer opportunities for career and skill development limit their performance motivation.

*“The amount of work we do does not commensurate with the salary, there is no scope of promotion in our job.” – ANM (50)*

*“There is lot of travel that we have to do but without any extra allowance.”- ANM (53)*

The workers seemed dissatisfied with the salary stating that that it is too less compared to the service they render. They also reported wide inter-state variations in remuneration of health workers of the same cadre. Travel to remote locations consumed lot of their time, efforts and money. Some CHWs also reported irregular payment of salary which was frustrating.

Majority of participants said they had worked for several years without being promoted and this was mentioned as an important factor for dissatisfaction. There were situations where senior experienced health workers earn more or less equal compared to those who were employed in recent years. This raises a climate of distrust and dissatisfaction among peers.

*“There are security concerns when we travel to remote hard-to-reach locations especially with the ladies, but there is nobody whom we can complain to”- ANM (15)*

The health workers looked worried regarding issues around security while travelling to remote difficult areas alone. Some of them also recalled instances of misbehavior by local residents. However they went on saying that they have to live with it as nobody is going to listen to them.

Poor personal health due to the work profile has been cited as a reason for low motivation by some. The nature of the job which includes daily travel to remote rural locations is a challenging task and puts burden on general health.

#### Family and community level

The CHWs expressed concern that they are not able to fulfil their family roles and spend time with them due to long hours of work.

*“Sometimes they call us on Sundays and holidays which causes conflicts in our family, and rightly so. I am not able to fulfill the daily chores and spend time with my family.”- ASHA (46)*

There is lack of family support because of long hours of work. Daily work-related travel is considered unsafe and inconvenient for women. They are also blamed for neglecting their children. Due to long working hours they are not able to fulfil their daily roles in the family and spend time with them.

#### Health system level

At the health system level, poor supply of logistics, non-involvement of CHWs in planning of service delivery and poor supportive supervision were identified as the key de-motivators.

CHWs reported poor logistic support including tools and supplies to do their work (for example, weighing scales, Blood Pressure apparatus, registers, notebooks, job aids, travel support etc.). They commented that damaged scales and instruments were not replaced, the government supply of medicines and consumables was depleted.

*“Very often what I am asked to do and what people [community] want from me is different. Whatever issues I raise on behalf of the community during the monthly meetings are not addressed.”- ASHA (06)*

The health workers sought their active involvement in planning of health service delivery rather than just being asked to follow the orders. Some CHWs mentioned that many times they are asked to do something by the higher authorities which is not liked by the community, but they have to do it because they cannot deny. This weakens community support for the work they do.

*“The supervisor does not listen to the difficulties we face. He is more concerned about the registers and reports. Even he is right because he is being asked to do this.”- ANM (12)*

*“The community recognizes our service, but supervisor never appreciates our work”- ASHA (04)*

Most CHWs viewed supervision as an opportunity to gain new knowledge and improve performance through problem solving and on-site instructions. However supervision is not carried out as intended. The CHWs reported lack of supportive supervision and lack of appreciation by the supervisors for their work. The supervisor has an attitude of fault finding and mostly concerned with timely submission of reports and filling of registers rather than improving the quality of community service delivery. Many CHWs complained that they never received any feedback from supervisory visits. They suggested sharing of feedback about issues identified during supervision so that they can be rectified.

## Discussion

The median (IQR) motivation score of health workers was 61 (58–64) out of a maximum score of 92. Age and training in the past 12 months were found to be significantly associated with motivation. Job burnout, poor personal health, job insecurity and less career development opportunities were individual level de-motivators, whereas not being able to fulfil family roles and poor supportive supervision were environmental factors for poor motivation. Love for work and financial incentives were individual level motivators. Community support and recognition, organizational commitment and pride, regular training were identified as environmental level motivators.

Motivation of health workers is key to providing good quality and accessible health care to population and achieving Sustainable Development Goals, especially in rural communities [8]. The results of this study could be useful especially in a setting like India where human resource is a major challenge. Measuring motivation is essential to address factors affecting motivation and design effective human resource strategies and policy decisions.

According to this study, although the workers were satisfied with the health services being provided by them and regarded them as essential, they felt that they did not get ample opportunities for skill and career development. A systematic review showed that health workers take pride and are motivated when they feel they have the opportunity to progress in their career [29]. Sundararaman et al. was also of the view that apart from monetary compensation, workforce management and continuous professional development are key approaches to retaining skilled health workers in rural India [14].

Another important finding was that those who had attended some form of training in the preceding 12-months had higher motivation scores when compared to those who had never attended any training. Previous studies have also shown that continuous in-service and refresher trainings could be a motivating factor for health workers [29–31]. This study supports continuous in-service and periodic refresher training as a source of both skills and motivation. Training increases self-confidence and morale of health professionals thereby affecting quality of care significantly [32]. Further studies are required to explore the reasons behind it.

Young age was associated with higher motivation scores similar to previous studies in the literature wherein middle aged health workers (35 and 54 years) reported lower levels of motivation [33]. This could be related to workload or the difficulty and complexity of the work which is usually greater for middle aged and elderly workers (who are often in senior-level positions) and also probably because of the dynamism and spirit of the young workers.

Family objection was a major motivational barrier to performance in job. Previous studies have found families as a source of discouragement [34–36] although some studies have found them as a source of positive support for CHWs [37].

The study showed that although salary and financial incentives are important, managers and policy makers should recognize non-financial motivators such as interpersonal relations, family support, skill and career development opportunities. Alternative income-generating activities such as cheap loans, opportunities for career advancement and professional development such as training and supportive supervision and non-monetary substitutes for remuneration such as transportation and supplies can address the financial needs [38]. Other studies have also shown that although financial incentives are important, they are not sufficient enough to improve health workers’ performance [13, 39]. In another study looking at driving factors of motivation for government doctors in India, more importance was given to intrinsic factors than extrinsic factors. The study showed that job security was the most important factor related to motivation, closely followed by interesting work and respect and recognition [20].

Recognition by the community was cited as being one of the most important motivating factors for health workers similar to other studies [6–38, 40–42]. Workers reported that they were encouraged by the nature of their work, being useful to society and taking care of people [40].

A comprehensive literature review on human resources in health in India has called for a national policy on human resources. The public sector will need to redesign a package of monetary and non-monetary incentives to encourage qualified health workers to work in rural and remote areas. It also suggested reorientation of education and training of health workers and improvement of provider quality through systems of continuing education, accreditation and regulation [15].

### Strengths and limitations

We employed a mixed method approach wherein in-depth interview responses verified the survey responses, thereby enhancing the generalizability of the study outcomes. Consolidated Criteria for Reporting Qualitative Research (COREQ) guidelines were adhered to while reporting the results of the qualitative component [27]. Nevertheless, there are three major limitations in this study. Firstly, it does not link motivation to service delivery in order to establish any possible causal link as this was beyond the scope of the current research. Secondly, it was possible that respondents could have been tempted to give high scores, thus biasing the results. Thirdly, the motivation scale used in this study has not been used earlier in India, although it has been used to assess motivation levels in health workers in similar developing settings.

## Conclusion

The managers and policy makers should recognize the importance of non-financial motivators such as interpersonal relations, family support, skill and career development opportunities. Frequent supervision and continuous training is essential to maintain high levels of motivation. Policy-makers and program implementers can use the various sources of motivation as a guide to design incentive structures and not just consider organizational level as a source of motivation to ensure the sustainability of CHW programs.

## Abbreviations

ANM, Auxiliary Nurse Midwife; ANOVA, analysis of variance; ASHA, Accredited Social Health Activist; AWC, Anganwadi Centre; AWW, Anganwadi Worker; CHC, Community Health Centre; CHW, Community Health Worker; IQR, interquartile range; MDGs, Millennium Development Goals; PHC, Primary Health Centre; SDGs, Sustainable Development Goals

## Additional file

Additional file 1: Questionnaire file. Motivation scale to assess levels of motivation of community health workers. It is a 23-item questionnaire with answers given on an agreement scale of 1 to 4 (1 = strong disagreement, 4 = strong agreement). Reverse coding was done for negative questions before analysis. The scale for negatively worded question was 1 (strong agreement) to 4 (strong disagreement). The tool had eight major constructs: general motivation, burnout, job satisfaction, intrinsic job satisfaction, organizational commitment, conscientiousness, timeliness and personal issues. (DOCX 15 kb)
