# Supplementary material for: Measuring and understanding motivation among community health workers in rural health facilities in India-a mixed method study
Source: BMC Health Serv Res. 2016 Aug 9;16:366. doi: 10.1186/s12913-016-1614-0 (PMC4977615; doi:10.1186/s12913-016-1614-0)
Supplement: Additional file 1: — Questionnaire file. Motivation scale to assess levels of motivation of community health workers. It is a 23-item questionnaire with answers given on an agreement scale of 1 to 4 (1 = strong disagreement, 4 = strong agreement). Reverse coding was done for negative questions before analysis. The scale for negatively worded question was 1 (strong agreement) to 4 (strong disagreement). The tool had eight major constructs: general motivation, burnout, job satisfaction, intrinsic job satisfaction, organizational commitment, conscientiousness, timeliness and personal issues. (DOCX 15 kb) [file 12913_2016_1614_MOESM1_ESM.docx]

**Motivation scale to assess levels of motivation of community health workers**

| **Category** | **Description of item** | **Score (1-4)** |
| --- | --- | --- |
| **General**  **Motivation** | I feel motivated to work hard |  |
|  | Only do this job to get paid |  |
|  | I do this job as it provides long-term security for me |  |
| **Burnout** | *I feel emotionally drained at the end of the day |  |
|  | *Sometimes when I get up in the morning, I dread having to face another day at work |  |
| **Job satisfaction** | Overall, I am very satisfied with my job |  |
|  | I am satisfied with my colleagues in my work |  |
|  | I am satisfied with my supervisor |  |
| **Intrinsic job satisfaction** | I am satisfied with the health services being provided by me |  |
|  | I feel that the services being provided by me are essential |  |
|  | I get ample opportunities for career and skill development |  |
| **Organization commitment** | I am proud to be working for this health facility |  |
|  | I feel very committed to this health facility |  |
|  | This health facility really inspires me to do my very best on the job |  |
| **Conscientiousness and self-efficacy** | I can rely on my colleagues at work |  |
|  | I always complete my tasks efficiently and correctly |  |
|  | Do things that need doing without being asked or told |  |
| **Timeliness** | I am punctual about coming to work |  |
|  | *I am often absent from work |  |
|  | It is not a problem if I sometimes come late for work/on leave |  |
| **Personal issues** | *I suffer from health related problems due to the work profile |  |
|  | *I feel difficulty in doing field activities |  |
|  | *My work affects my duties towards my family |  |

*The scale for these negatively worded questions was reverse coded so that 1 was ‘strong agreement’ and 4 ‘strong disagreement’. Thus, a high score shows disagreement with a negative statement and is therefore suggestive of higher motivation.
